# Diphtheria

**Published:** 1884-11

**Authors:** E. B. Nash


### DIPHTHERIA.

Dr. E. B. Nash.

(Reported to Central New York Homœopathic Medical Society, March 20th, 1884.)

Case I.—Carrie Bostwick, aged twelve years, very fleshy and full-faced; ruddy. Symptoms when first visited were: General heat, very high temperature, and quick pulse; great headache, with very red face; throbbing carotids and pains in back and limbs; throat very red; patches on both tonsils, which were so swollen as to fill the throat almost full.

Bell.$^{30}$ in solution once in two hours until next day, when all the symptoms were not in the least abated.

Examination of the throat revealed the membrane greatly increased on both sides and a heavy ring of the same around the uvula, the end of which looked œdematous, like a bag of water, and much elongated. She then described the pains in the throat as stinging pains, very sharp, and running up into both ears, so that she would cry out and clap her hands to the ears when they occurred. She could not lie upon the back at all because she could not breathe, but lay with her face downward on the edge of the pillow over a spittoon, letting the saliva, which was profuse, as well as a discharge from the nose, run into it; breath so offensive the windows were left up in her room.

Apis$^{30}$ in solution once in two hours.

Improvement very marked at the next visit next day, particularly the pains and restlessness, all the other objective symptoms following in their wake in due time. Under this, the homœopathic, remedy the case recovered, except that a profuse epistaxis ensued which set in at night during convalescence. It bled three nights, when, on account of the readiness of the blood to form *large clots, "which hung down like icicles from the nose,"* Merc. sol.$^{2c}$ was given, which wound up the bleeding.

Two weeks later she found herself not able to read her reading book as near as usual; had to hold it off a long way, and finally could hardly read at all (paralysis of accommodation).

Gels.$^{30}$ corrected this in a few days, and henceforth she remains well.

Case II.—Her sister, aged eight years, same temperament, but little darker hair, eyes, and complexion, was taken a few days after. Not being quite able to recognize the right remedy in this case, and because Apis worked so nicely in the other, Apis was given for the first remedy.

Next day no improvement, and careful examination revealed as follows: *Disease commenced apparently in the nose and extended to right tonsil, thence to left, and the right one particularly swelling enormously*, extending to the cellular tissues so that the right side of neck was swollen clear out beyond the jaw and began to turn purple. This child also was not able to lie upon the back at all on account of not being able to breathe. Not so much discharge from mouth and none from the nose. When she wakes out of her short, restless naps, "*awakes kicking and screaming and very cross.*" *Breath horribly offensive.*

Lycop.$^{1m}$, B. & T., one dose, dry, on tongue, one dissolved in three teaspoonfuls of cold water—one spoonful once an hour, followed by Sac. lac.

Improvement prompt, rapid, and perfect. Nothing else was needed. This child had had several scrofulous swellings in the neck during her earlier childhood and I expected to get an opening in this blue swelling above described.

Two others were attacked in the family—one brother and the father—but both lightly, and Apis for the one and Phytol. for the other was all that was needed. Do not think either would have been serious had no medicine been given.

The house in which this family lived had a cellar under it in which water stands every spring, it being on low, marshy ground, and this last spring had been neglected, so that a bad smell came from it. That this was genuine diphtheria none will doubt.

Note that *no "whiskey in excess"* (Eugene Stoke, counsellor), gargles, alternated remedies, brandy, or quinine was necessary, nor ever are, provided the simillimum is found and properly used.

